# Impact of COVID-19 pandemic on carbapenem-resistant Enterobacterales incidence in the South-East Asia region: an observational study

**DOI:** 10.1017/ash.2023.477

**Published:** 2023-11-15

**Authors:** Kyaw Zaw Linn, Stephanie Sutjipto, Oon Tek Ng, Jeanette Teo, Benjamin Pei Zhi Cherng, Thean Yen Tan, Surinder Kaur Pada, Say Tat Ooi, Nares Smitasin, Koh Cheng Thoon, Xiaowei Huan, Partha Pratim De, Douglas Chan, Nancy Wen Sim Tee, Michelle Ang, Li Yang Hsu, Raymond Tzer Pin Lin, Tong Yong Ng, Rama Narayana Deepak, Tse Hsien Koh, Anucha Apisarnthanarak, Sasheela Ponnampalavanar, Indumathi Venkatachalam, Kalisvar Marimuthu

**Affiliations:** 1 National Centre for Infectious Diseases, Singapore, Singapore; 2 Tan Tock Seng Hospital, Singapore, Singapore; 3 Lee Kong Chian School of Medicine, Nanyang Technological University, Singapore, Singapore; 4 National University Hospital and National University Health System, Singapore, Singapore; 5 Singapore General Hospital, Singapore, Singapore; 6 Duke-NUS Medical School, National University of Singapore, Singapore, Singapore; 7 Yong Loo Lin School of Medicine, National University of Singapore and National University Health System, Singapore, Singapore; 8 Changi General Hospital, Singapore, Singapore; 9 Ng Teng Fong General Hospital, Singapore, Singapore; 10 Khoo Teck Puat Hospital, Singapore, Singapore; 11 KK Women’s and Children’s Hospital, Singapore, Singapore; 12 Saw Swee Hock School of Public Health, National University of Singapore and National University Health System, Singapore, Singapore; 13 Singapore Centre for Environmental Life Sciences Engineering, National University of Singapore, Singapore, Singapore; 14 National Public Health Laboratory, Singapore, Singapore; 15 Sengkang General Hospital, Singapore, Singapore; 16 Thammasat University Hospital, Bangkok, Thailand; 17 University of Malaya Medical Centre, Kuala Lumpur, Malaysia

## Abstract

The COVID-19 pandemic led to an initial increase in the incidence of carbapenem-resistant Enterobacterales (CRE) from clinical cultures in South-East Asia hospitals, which was unsustained as the pandemic progressed. Conversely, there was a decrease in CRE incidence from surveillance cultures and overall combined incidence. Further studies are needed for future pandemic preparedness.

## Introduction

The COVID-19 pandemic brought about unprecedented challenges to hospital management with the sudden influx of patients and rapidly evolving situations causing changing and occasionally conflicting infection control recommendations from international authorities.^1^ While some infection control measures against COVID-19 such as donning masks in inpatient areas, and restriction of visitor numbers and hours, may also reduce the transmission of carbapenem-resistant Enterobacterales (CRE), other resource prioritization measures for COVID-19 such as prioritizing personal protective equipment for COVID-19, reserving single rooms for COVID-19, pausing the routine infection prevention and control (IPC) surveillance may increase the CRE incidence. There have been reports of a decline in multidrug-resistant organisms transmission attributed to COVID-19 pandemic, but the reports were either limited to a single hospital or campus,^2,3^ hence, the findings may not be conclusive and generalizable. In this observational study, we explored the incidence of CRE in South-East Asia hospitals before and during the COVID-19 pandemic.

## Method

This is a retrospective review of the incidence of CRE data from eight hospitals in the South-East Asia region: six in Singapore, one in Thailand, and one in Malaysia. Four hospitals are academic medical centers, while the remaining four are acute care hospitals. Three hospitals have a bed capacity of over 1,500 beds, two have between 1,000 and 1,200 beds, and the remaining three have between 400 and 800 beds. Monthly incident CRE cases and hospital patient-days data were collected between January 2019 and March 2021. All hospitals except one could provide separate data on CRE incident cases from clinical and surveillance cultures. All three countries reported their first COVID-19 case in January 2020, hence, we considered the period from January 2019 to January 2020 as the pre-pandemic period, and from February 2020 to March 2021 as the pandemic period.

### Statistical analysis

We analyzed the impact of COVID-19 pandemic on CRE clinical cultures, surveillance cultures, and a combined analysis of CRE clinical and surveillance cultures (all CRE). Data from participating sites were analyzed collectively (combined analysis) and individually.

An interrupted time series analysis was performed using the ordinary least-squared regression-based Newey-West model on STATA^4^ to detect any significant changes in the CRE incidence per 1,000 patient days between pre-pandemic and pandemic period. We used the Cumby-Huizinga test (actest command in STATA) to assess autocorrelation. The lag value was included in the model to account for autocorrelation when the *p*-value was less than 0.05.

## Result

A total of 2,562 incident cases of CRE in pre-pandemic and 2,323 incident cases of CRE during the COVID-19 pandemic were included in the analysis. The patient days were 2,866,737 during the pre-pandemic period and 2,839,913 during the pandemic (Supplementary Table S1). Supplementary Table S1 shows the detailed number of the incident cases of CRE from surveillance and clinical cultures and patient days before and during pandemic.

**Figure 1.**
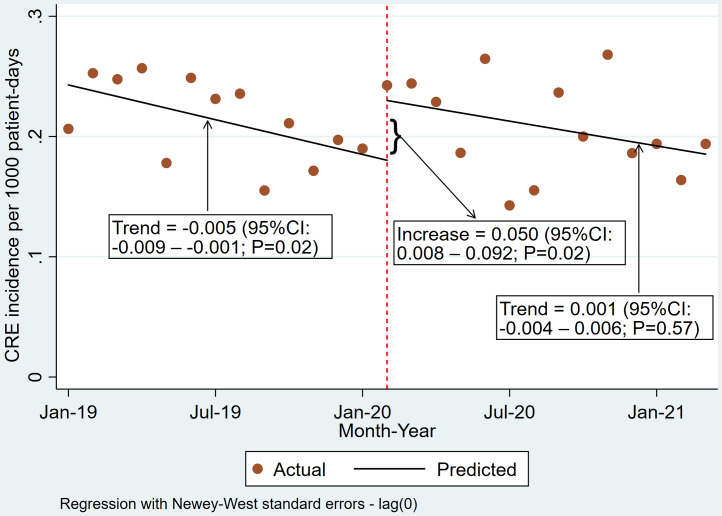
Incidence of CRE from clinical cultures at all participating sites.

In the combined analysis of all sites, we observed a significant step change increase in the incidence of clinical cultures positive for CRE at the onset of the pandemic, with a rate of 0.050 cases per 1,000 patient days (95% CI: 0.008–0.092; *P* = 0.02) (Figure 1). However, there was no significant difference in the trend during the pandemic compared to the pre-pandemic period, with a rate of 0.001 (95% CI: −0.004 to 0.006; *P* = 0.57). The step change and trend change of individual hospitals are described in Table 1.

**Table 1.** Incidence rate trends of carbapenem-resistant Enterobacterales from clinical cultures, surveillance cultures, and combined clinical and surveillance cultures across the Asia-pacific hospitals from interrupted time series analysis

Hospital	Monthly change in CRE incidence in pre-pandemic period		Step change in CRE incidence rate at the start of the pandemic period		Monthly change in CRE incidence trend during the pandemic compared to the pre-pandemic period	
	Incidence rate (95%CI)	*P* value	Incidence rate (95%CI)	*P* value	Incidence rate (95%CI)	*P* value
Incidence of CRE from clinical cultures						
Hospital 1	−0.005 (−0.016 to 0.005)	0.33	−0.072 (−0.179 to 0.034)	0.17	0.010 (−0.004 to 0.025)	0.14
Hospital 2	0.008 (0.001 to0.016)	0.03	−0.068 (−0.171 to 0.035)	0.19	−0.012 (−0.026 to 0.003)	0.11
Hospital 3	−0.0005 (−0.013 to 0.012)	0.94	0.025 (−0.066 to 0.117)	0.57	−0.001 (−0.023 to 0.020)	0.90
Hospital 4	−0.003 (−0.015 to 0.009)	0.65	0.076 (−0.098 to 0.250)	0.38	−0.006 (−0.027 to 0.015)	0.55
Hospital 5	−0.036 (−0.054 to −0.017)	0.001	0.139 (−0.012 to 0.290)	0.07	0.042 (0.020 to 0.064)	0.001
Hospital 6	0.011 (0.002 to 0.019)	0.01	0.046 (−0.049 to 0.142)	0.32	−0.023 (−0.036 to −0.011)	0.001
Hospital 7	Not available					
Hospital 8	0.003 (−0.011 to 0.018)	0.62	0.178 (−0.029 to 0.385)	0.09	−0.021 (−0.043 to 0.002)	0.08
All hospitals combined	−0.005 (−0.009 to −0.001)	0.02	0.050 (0.008 to 0.092)	0.02	0.001 (−0.004 to 0.006)	0.57
Incidence of CRE from surveillance cultures						
Hospital 1	0.011 (−0.016 to 0.037)	0.43	0.265 (−0.537 to 1.067)	0.50	−0.027 (−0.124 to 0.069)	0.56
Hospital 2	−0.0003 (−0.008 to 0.008)	0.94	0.191 (0.076 to 0.306)	0.002	−0.018 (−0.033 to −0.003)	0.02
Hospital 3	0.009 (−0.057 to0.076)	0.77	−0.362 (−1.053 to 0.329)	0.29	0.010 (−0.067 to 0.088)	0.78
Hospital 4	0.008 (−0.007 to 0.023)	0.29	0.039 (−0.130 to 0.208)	0.64	−0.032 (−0.051 to −0.014)	0.002
Hospital 5	0.0005 (−0.016 to 0.017)	0.96	−0.416 (−0.676 to −0.156)	0.003	0.022 (−0.008 to 0.053)	0.15
Hospital 6	0.093 (0.046 to 0.139)	<0.001	−0.660 (−1.590 to 0.269)	0.16	−0.123 (−0.238 to −0.009)	0.04
Hospital 7	Not available					
Hospital 8	0.001 (−0.013 to 0.014)	0.89	0.077 (−0.080 to 0.234)	0.32	0.030 (0.012 to 0.048)	0.002
All hospitals combined	0.023 (0.011 to 0.035)	0.001	−0.178 (−0.347 to −0.010)	0.04	−0.026 (−0.043 to −0.009)	0.004
Incidence of CRE from combined clinical and surveillance cultures						
Hospital 1	0.009 (−0.024 to 0.042)	0.58	0.183 (−0.645 to 1.011)	0.65	−0.024 (−0.127 to 0.078)	0.63
Hospital 2	0.005 (−0.002 to 0.013)	0.16	0.114 (−0.009 to 0.237)	0.07	−0.022 (−0.038 to −0.006)	0.01
Hospital 3	0.005 (−0.069 to 0.078)	0.90	−0.348 (−1.074 to 0.377)	0.33	0.011 (−0.073 to 0.096)	0.79
Hospital 4	0.008 (−0.007 to 0.024)	0.29	0.047 (−0.139 to 0.233)	0.61	−0.036 (−0.055 to −0.016)	0.001
Hospital 5	−0.014 (−0.044 to 0.016)	0.35	−0.351 (−0.591 to −0.110)	0.01	0.039 (−0.005 to 0.083)	0.08
Hospital 6	0.093 (0.038 to 0.148)	0.002	−0.620 (−1.55 to 0.314)	0.18	−0.131 (−0.254 to −0.008)	0.04
Hospital 7	0.013 (−0.012 to −0.038)	0.28	0.404 (−0.036 to 0.844)	0.07	−0.072 (−0.125 to −0.019)	0.01
Hospital 8	0.004 (−0.016 to 0.025)	0.67	0.255 (0.018 to 0.491)	0.04	0.010 (−0.014 to 0.034)	0.41
All hospitals combined	0.019 (0.007 to 0.031)	0.003	−0.146 (−0.308 to 0.016)	0.076	−0.028 (−0.044 to −0.012)	0.001

Note. CRE, carbapenem-resistant Enterobacterales.

There was a significant step change decrease in the combined incidence of surveillance cultures positive for CRE at the beginning of the pandemic, with a rate of −0.178 cases per 1,000 patient days (95% CI: −0.347 to −0.010; *P* = 0.04) (Supplementary Figure S1). Furthermore, there was a significant overall decrease trend during the pandemic compared to the pre-pandemic period, with a rate of −0.026 (95% CI: −0.043 to −0.009; *P* = 0.004).

There was an initial decrease in the combined incidence of all CRE cultures at the beginning of the pandemic, with a rate of −0.146 (95% CI: −0.308 to 0.016; *P* = 0.08) (Supplementary Figure S2). This was followed by a significant overall decrease trend during the pandemic compared to the pre-pandemic period, with a rate of −0.028 (95% CI: −0.044 to −0.012; *P* = 0.001).

## Discussion

In this multicentered study in the South-East Asia region, we found an initial significant increase in the incidence of CRE from clinical cultures, which was not sustained as the pandemic progressed. Changing patient demographics could partially explain the initial rise in CRE incidence, for instance, the implementation of various government policies to postpone elective admissions and surgeries, and general public’s avoidance of healthcare facilities due to fear of COVID-19. Only seriously ill or long-stayer patients at higher risk of having CRE infection likely remained in hospitals during this period. Stabilization of patient case mix with possibly heightened IPC measures may explain the CRE clinical culture trend being similar to pre-pandemic levels.

During the pandemic, there was a significant decrease in CRE surveillance cultures and the overall number of CRE screening tests. This might be because fewer patients meet the criteria for CRE screening; for instance, fewer overseas travelers due to travel restrictions. Additionally, resource allocation for COVID-19 may have affected the hospitals’ CRE screening capacity. The enhanced IPC measures during the COVID-19 pandemic may genuinely reduce CRE colonization. Further investigations into these reasons could greatly facilitate future pandemic preparedness.

The inclusion of eight hospitals from three countries in the South-East Asia is the key strength of this study. This study also provides hypotheses for future studies and highlights that future pandemic response plans should incorporate strategies to maintain day-to-day IPC measures during a pandemic. Our study has some limitations. First, even though the first case of COVID-19 was discovered in January 2020, the factors that might have impacted the CRE incidence may have occurred before or after January 2020. Hence, the selection of the cutoff point may not have been accurate. However, we observed a sharp decline in patient days in February 2020 (Supplementary Figure S3), suggesting that the biggest impact of COVID-19 on hospital services may have been in February 2020. Additionally, we do not have the patient-level data on the factors that can influence the acquisition of CRE, such as age and comorbidities, as the change in the demographic and other patient-level data could have explained the change in the CRE trends between the two periods. Finally, we could only include the 8 hospitals in the Asia-Pacific region, mostly from Singapore, which may affect the generalizability of our findings.

In conclusion, the COVID-19 pandemic has had minimal impact on the incidence of CRE from clinical cultures, but a significant reduction was seen in the incidence of CRE from surveillance cultures. The effect was not uniform across the participating sites, and follow-up studies are needed to look into specific contributing factors to the observed trend in CRE across these hospitals.

## Supporting information

Linn et al. supplementary material 1 Linn et al. supplementary material

Linn et al. supplementary material 2 Linn et al. supplementary material

Linn et al. supplementary material 3 Linn et al. supplementary material

Linn et al. supplementary material 4 Linn et al. supplementary material

## Supplementary material

The supplementary material for this article can be found at https://doi.org/10.1017/ash.2023.477.
