# Supplementary figures and images for: Impact of COVID-19 pandemic on carbapenem-resistant Enterobacterales incidence in the South-East Asia region: an observational study
Source: Antimicrob Steward Healthc Epidemiol. 2023 Nov 15;3(1):e208. doi: 10.1017/ash.2023.477 (PMC10753475; doi:10.1017/ash.2023.477)

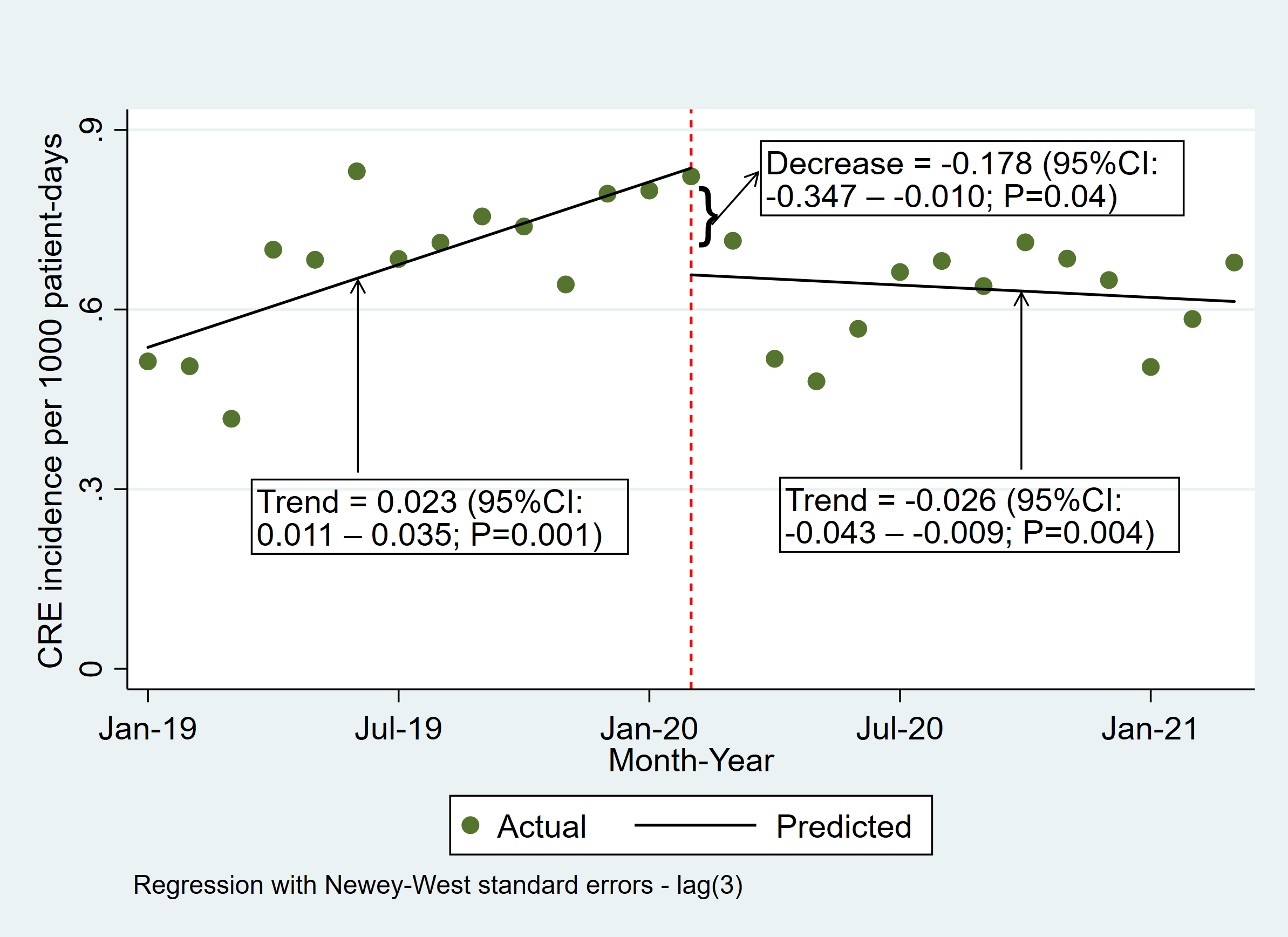

Supplement: Linn et al. supplementary material 1 — Linn et al. supplementary material [file S2732494X23004771sup001.tif]

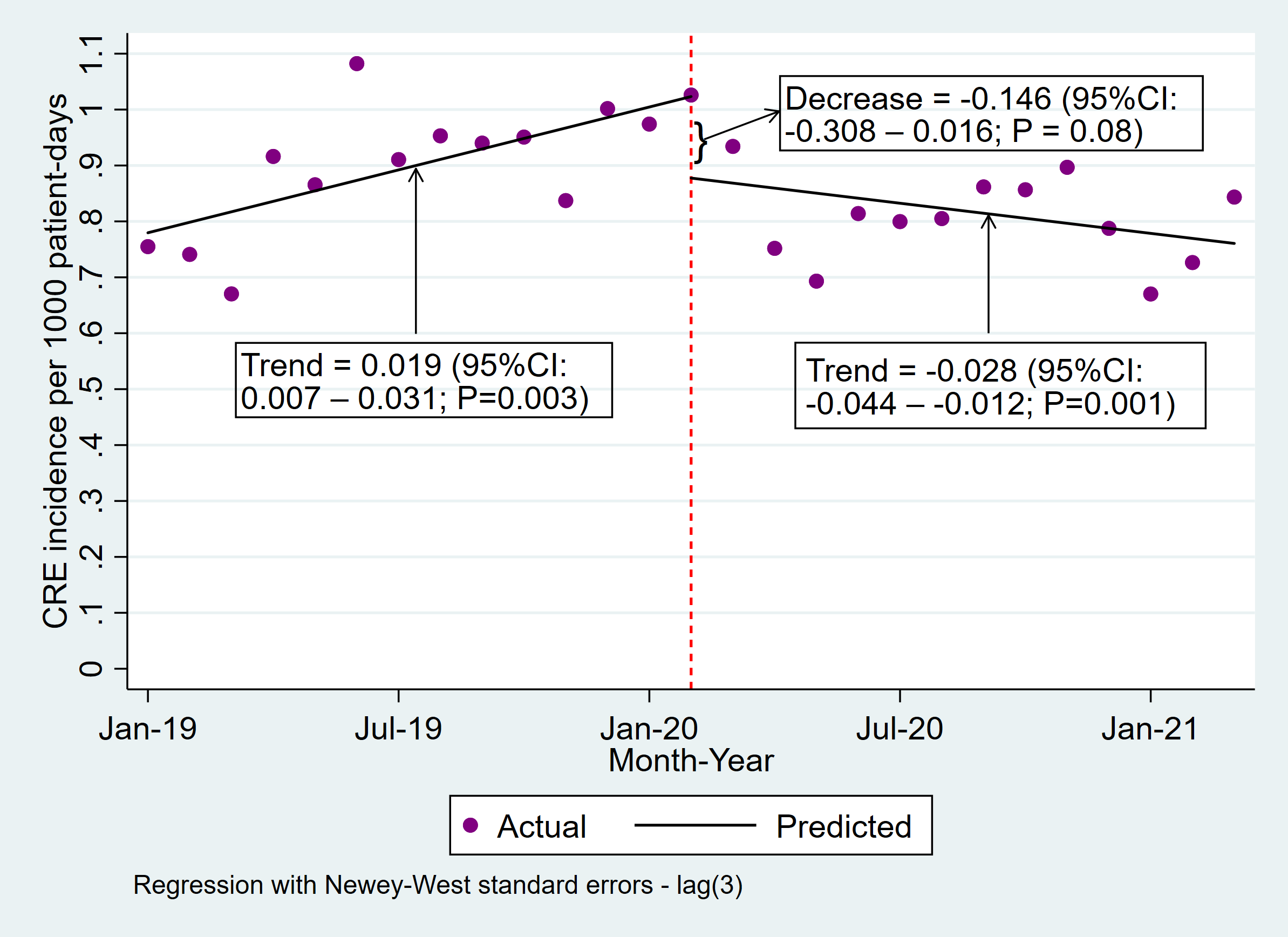

Supplement: Linn et al. supplementary material 2 — Linn et al. supplementary material [file S2732494X23004771sup002.tif]

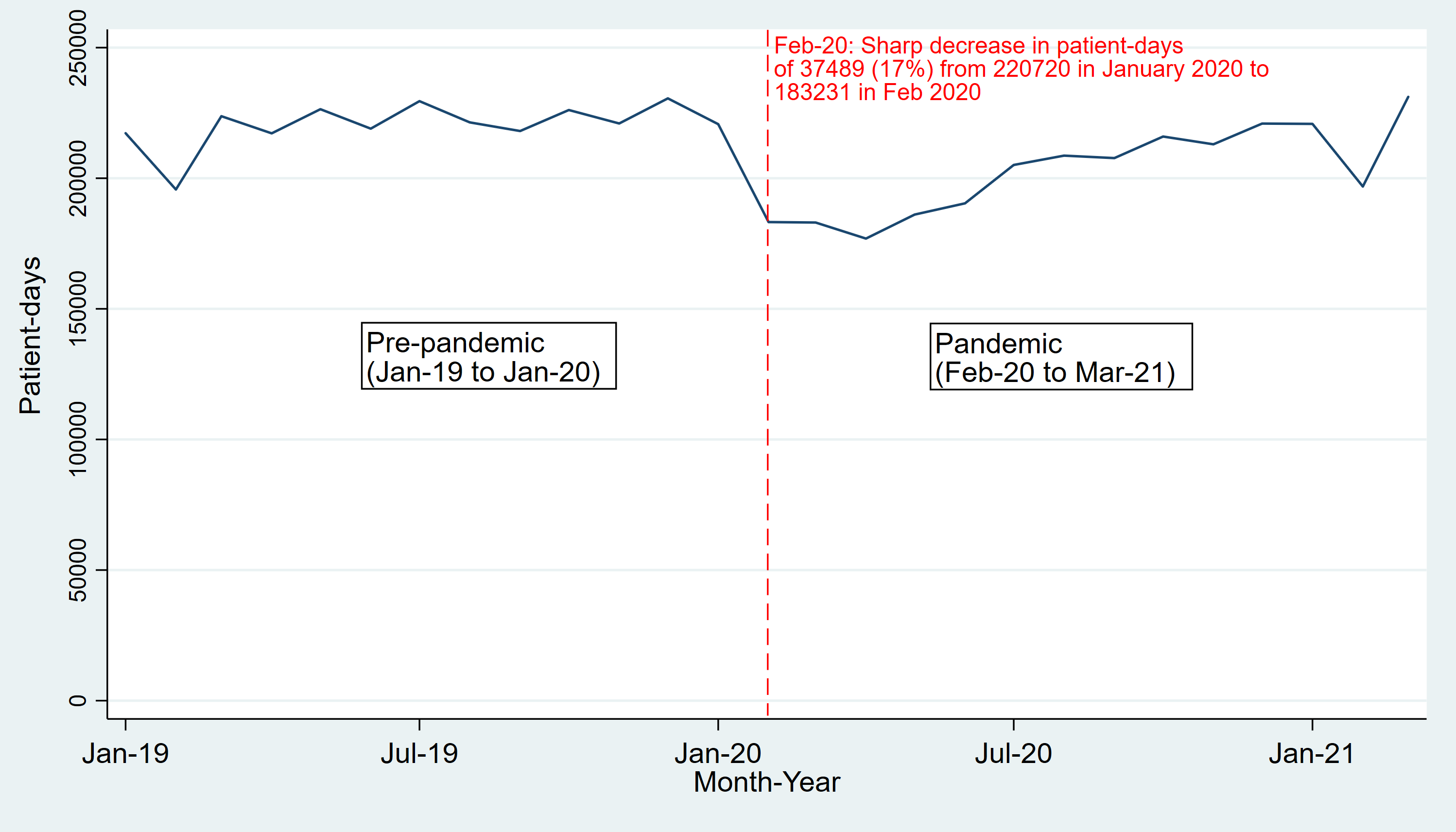

Supplement: Linn et al. supplementary material 3 — Linn et al. supplementary material [file S2732494X23004771sup003.tif]
