# Supplementary material for: Impact of COVID-19 pandemic on carbapenem-resistant Enterobacterales incidence in the South-East Asia region: an observational study
Source: Antimicrob Steward Healthc Epidemiol. 2023 Nov 15;3(1):e208. doi: 10.1017/ash.2023.477 (PMC10753475; doi:10.1017/ash.2023.477)
Supplement: Linn et al. supplementary material 4 — Linn et al. supplementary material [file S2732494X23004771sup004.docx]

**Appendix Table 1: Incident CRE cases and patient-days at participating sites**

| Variables | Pre-pandemic period | Pandemic period |
| --- | --- | --- |
| Incident cases of CRE from clinical cultures | 563 | 554 |
| Incident cases of CRE from surveillance cultures | 1785 | 1707 |
| Patient-days of 7 hospitals | 2,636,445 | 2,682,104 |
| Incident cases of CRE from combined clinical and surveillance cultures | 2562 | 2323 |
| Patient-days of 8 hospitals | 2,866,737 | 2,839,913 |
